# Review article: Scoping review of interventions that reduce mechanical restraint in the emergency department

**DOI:** 10.1111/1742-6723.14498

**Published:** 2024-10-03

**Authors:** Joseph Lee, Daiv J Lown, Patrick J Owen, Judith Hope

**Affiliations:** ^1^ Eastern Health Clinical School Monash University Melbourne Victoria Australia; ^2^ Eastern Health Emergency Medicine Program Melbourne Victoria Australia; ^3^ Mental Health and Wellbeing Program, Eastern Health Melbourne Victoria Australia; ^4^ Delmont Centre for Education and Research Melbourne Victoria Australia

## Abstract

**Objective:**

Mechanical restraints are known to be associated with many undesirable outcomes in clinical settings. Our objective was to examine the current literature to explore possible interventions that would reduce the use of mechanical restraints in the ED.

**Methods:**

In this scoping review, we searched online databases Embase, MEDLINE and Cochrane CENTRAL for any studies published between the databases from 1 January 2007 to 19 September 2023. Studies were included if interventions were hospital‐ or staff‐focused and reported measured outcomes before and after the introduction of the intervention. Risk of bias was assessed using the JBI Critical Appraisal Checklist for Cohort Studies.

**Results:**

The search strategy yielded 1937 studies across the three databases, of which 13 studies were extracted and included in the review. Interventions were categorised into four groups: provision of staff training, addition of a de‐escalation team, creation of a dedicated unit and introduction of an agitation scale only. Most of the studies saw reduction in restraint rates or time in restraints. Only the two studies that used an agitation scale as a stand‐alone intervention saw no significant reduction. Only one study had low risk of bias, whereas the remainder had high risk.

**Conclusions:**

Evidence supports further exploration of interventions that include: designing an agitation guideline; training staff in assessment, attitudinal and de‐escalation skills; addition of a crisis team; and environmental changes in the form of adding a dedicated clinical space. Although these strategies may reduce mechanical restraint in the ED setting, further high‐quality studies are needed before definitive conclusions may be drawn.

## Graphical abstract

Mechanical restraints are known to be associated with many undesirable outcomes in clinical settings. We examined the literature and found that several interventions haveworked to reduce mechanical restraint rates in emergency departments.

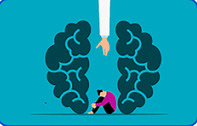

**Key findings**

- Staff education and attitudinal change.
- Addition of a trained crisis team.
- Environmental modifications.

## Background

Mechanical restraint refers to the practice of using physical devices to restrict a patient's movements typically in response to behaviours of concern.^1^ In clinical practice, mechanical restraint is most commonly employed in emergency and psychiatric settings, and spans both adult and paediatric population groups. Uses of mechanical restraint may include managing potentially harmful behaviours associated with drug/alcohol intoxication, mental health disorders or neurological conditions (e.g. dementia), as well as achieving stability (particularly in paediatric population groups) to enable procedures. Although the practice was intended to protect the patient and staff, the use of mechanical restraints is problematic in nature with potentially widespread consequences.

Mechanical restraint use is associated with many adverse physical and psychological outcomes for patients, which may differ in severity based on duration and nature of restraint. Physical issues include limb injuries, pneumonia, deep venous thrombosis and other major events, including death because of trauma asphyxia.^2^, ^3^, ^4^, ^5^ People who are subjected to forced immobilisation may experience extreme psychological distress and worsening of mental health conditions, which can be severe.^6^ These experiences can have enduring effects on a person's well‐being and contribute to persistent trauma and mistrust towards healthcare systems.^7^, ^8^

The practice of mechanical restraint also has a detrimental impact on staff. Those who utilise mechanical restraints may be subjected to emotional distress. This can affect staff morale,^9^ and contribute to moral distress^10^ and burnout.^9^

Consumer, clinical and government organisations have advocated for the reduction and complete cessation of mechanical restraint use.^11^, ^12^, ^13^, ^14^ In the local context of our work, the Victorian Royal Commission into Mental Health has recommended the complete cessation of mechanical restraints by 2031.^15^

Progressive efforts have been made to create reduction in restrictive practices. The majority of research into evidence‐based interventions has been conducted in the psychiatric setting.^16^ A range of approaches have been explored.

Existing strategies are diverse, and include both frameworks and specific interventions. An example of a restraint reduction conceptual framework is the *Six Core Strategies to Reduce the Use of Seclusion and Restraint*, comprising of leadership towards organisational change, use of data to inform practice, workforce development, use of seclusion/restraint prevention tools, consumer roles in inpatient settings and post‐incident support and review.^17^ The *Safewards* initiative, an intervention originating in England after the review of decades of literature,^18^, ^19^ has been deployed in Victoria, Australia and other locations in psychiatry inpatient settings and more recently in ED. *Safewards* utilises 10 key interventions on mental health wards to engage with and empower consumers (Table 1).^20^

**TABLE 1:** Ten *Safewards* interventions^20^

Know each other
Clear mutual expectations
Mutual help meeting
Calm down methods
Bad news mitigation
Soft words
Talk down
Reassurance
Discharge messages
Positive words

Some research has been conducted in ED settings. In particular, interventions such as choice of pharmacological agent for management of behaviours of concern have been well researched and established protocols for this already exist. Hence, this review sets out to explore what staff‐ and hospital‐focused interventions have been conducted to reduce restrictive interventions in an ED setting.

## Methods

This literature review was conducted according to PRISMA guidelines for a scoping review.^21^ Our choice to conduct a scoping review, rather than systematic review, was borne from our overarching aim to investigate research conducted to reduce restrictive interventions in an ED setting.^22^ The protocol was registered with the Open Science Framework ahead of conducting the searches (https://osf.io/2b5w7).

### Search strategy

The articles were searched using three online databases from 1 January 2007 to 19 September 2023: Embase, Ovid MEDLINE and the Cochrane Library. We included studies published after 1 January 2007 to capture contemporary practice. A search strategy was established for each database to adhere to the inclusion and exclusion criteria. Table 2 outlines the search terms used for each of the databases.

**TABLE 2:** Search strategy

Source	Search strategy
Embase via Ovid	[#1] exp emergency health service/ [#2] emergency health service/ [#3] ((emergency adj3 (department* or medicine or care or ward* or room* or unit* or service* or setting* or hospital* or health service*)) or "accident and emergency" or casualty department* or A&E or ER or ED or EMS).ti,ab,kf. [#4] #1 or #2 or #3 [#5] ((mechanical or physical) adj3 restraint*).mp. [#6] exp physical restraint/ [#7] (restraint* or ((management adj3 (agitation or aggress*)) or "leather restrain*" or "coercive measure")).mp. [#8] containment measure*.mp. [#9] (patient* adj3 (aggress* or restrain*)).mp. [#10] #5 or #6 or #7 or #8 or #9 [#11] (intervention* or "organisational intervention*" or "education* intervention*").mp. [#12] #4 and #10 and #12 [#13] limit #12 to yr="2007 ‐Current"
Ovid MEDLINE	[#1] exp emergency health service/ [#2] emergency health service/ [#3] ((emergency adj3 (department* or medicine or care or ward* or room* or unit* or service* or setting* or hospital* or health service*)) or "accident and emergency" or casualty department* or A&E or ER or ED or EMS).ti,ab,kf. [#4] #1 or #2 or #3 [#5] ((mechanical or physical) adj3 restraint*).mp. [#6] exp physical restraint/ [#7] (restraint* or ((management adj3 (agitation or aggress*)) or "leather restrain*" or "coercive measure")).mp. [#8] (containment measure*).mp. [#9] (patient* adj3 (aggress* or restrain*)).mp. [#10] #5 or #6 or #7 or #8 or #9 [#11] (intervention* or "organisational intervention*" or "education* intervention*").mp. [#12] #4 and #10 and #11 [#13] limit #12 to yr="2007 ‐Current"
CENTRAL	[#1] MeSH descriptor: [Emergency Service, Hospital] explode all trees [#2] MeSH descriptor: [Emergency Medical Services] this term only [#3] (((emergency NEAR/3 (department* OR medicine OR care OR ward* OR room* OR unit* OR service* OR setting* OR hospital* OR health service*)) OR "accident and emergency" OR casualty department* OR A&E OR ER OR ED OR EMS)):ti OR (((emergency NEAR/3 (department* OR medicine OR care OR ward* OR room* OR unit* OR service* OR setting* OR hospital* OR health service*)) OR "accident and emergency" OR casualty department* OR A&E OR ER OR ED OR EMS)):ab [#4] #1 OR #2 OR #3 [#5] ((mechanical OR physical) NEAR/3 restraint*) [#6] MeSH descriptor: [Restraint, Physical] this term only [#7] (restraint* or ((management NEAR/3 (agitation or aggress*)) or leather restrain* or "coercive measure")) [#8] bedrail* or bedchair* or containment measure* [#9] (patient* NEAR/3 (aggress* or restrain*)) [#10] #5 OR #6 OR #7 OR #8 OR #9 [#11] #4 AND #10 with Publication Year from 2007 to 2023, in Trials

### Inclusion and exclusion criteria

Included studies were those published in full (i.e. conference abstracts excluded) within English language peer‐reviewed journals (i.e. grey literature excluded). Other criteria followed the Population, Intervention, Comparator, Outcome (PICO) framework.^23^ *Population*: patients of any hospital ED, regardless of age (i.e. adult or paediatric) or condition (i.e. psychiatric inpatient population groups were included). *Intervention*: any organisational‐level strategy (e.g. environmental changes, system and organisational adjustments, and educational programs). Individual‐level interventions, such as those focusing on individual patient pharmacotherapy, were excluded given our aim to identify organisational‐level strategies. *Comparator*: any. *Outcome*: any outcome associated with mechanical restraint (e.g. use/duration of restraint, use/duration of seclusion, length of stay and rates of codes).

### Data collection

All studies across the three databases were imported into Covidence and duplicates were automatically removed. During title/abstract, full‐text screening and extraction, two independent authors (DJL and JL) assessed all articles and discrepancies were resolved by a third independent author (JH). Extracted information included publication information (e.g. author, year, title and journal), design of study, setting country, population, sample size, intervention, outcome measures, duration of study period and key results.

One author (PJO) used the JBI Critical Appraisal Checklist for Cohort Studies to determine the risk of bias for each included study.^24^ The tool includes 11 questions answered with yes, no, unsure or not applicable. Overall risk of bias was interpreted as high (any response was no), moderate (any response was unclear) or low (all responses were yes).^24^

## Results

**Figure 1:**
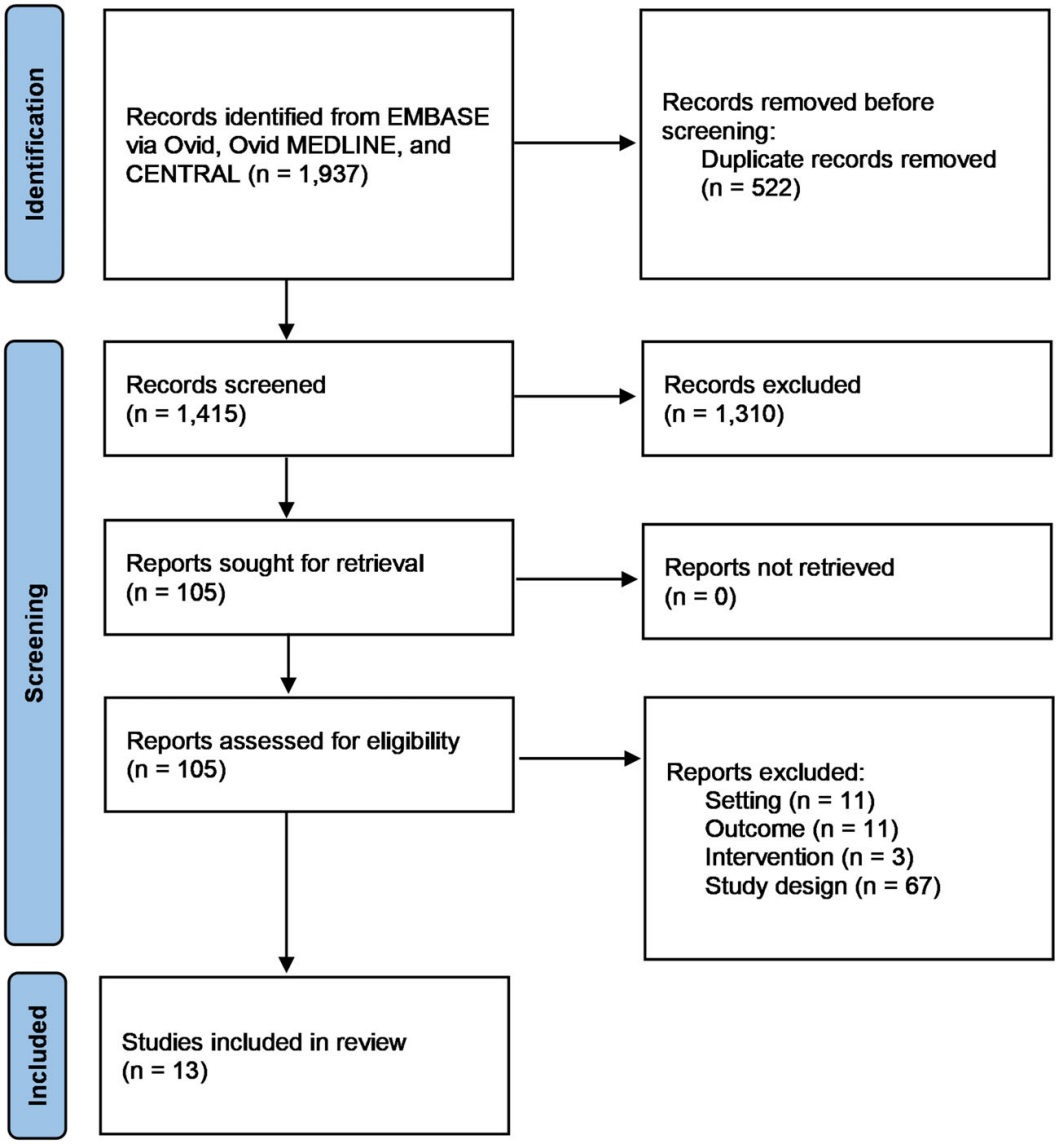
PRISMA flow chart.

The literature search yielded 1937 studies across the three databases. Duplicates (522) were removed automatically. Among the 1415 studies left, 105 studies were retrieved for full‐text review. An additional 92 studies were excluded because of wrong methodology, such as objectives, setting and outcome measures. At the end of the screening process, 13 studies were selected for extraction (Table 3). Figure 1 demonstrates the data collection process.

**TABLE 3:** Data extraction table

Study	Design	Setting and country	Population	Sample size	Intervention	Outcome measures, duration of study period	Key results
Braitberg *et al*.^33^	Pre/post‐intervention study	General Hospital ED Australia	All Behavioural Assessment Unit (BAU) admissions Pre‐BAU median age 34 *vs*. post‐BAU median age 33	2379 patients admitted post‐BAU *vs*. 3047 patients pre‐BAU	Introduction of BAU	Primary: length of stay(LOS) in ED Secondary: time to clinician, crisis team activation rates (Code Grey), restrictive intervention rates 12 months post BAU with a matched cohort from 2 years prior	Decrease ED LOS from 328 to 180 min Decrease of total number of ‘Code Grey’ from 370 patients having 538 ‘Code Grey’ to 259 patients having 349 ‘Code Grey’ Mechanical restraint rate reduced from 9.0% (275 patients out of 3047) to 6.6% (156 patients out of 2379) *P* < 0.001
Browne *et al*.^25^	Pre/post‐intervention study	General Hospital Australia	All Psychiatric Assessment and Planning Unit (PAPU) admissions	Unspecified	Introduction of PAPU	LOS in ED, one‐to‐one nursing duration, mechanical restraint rate, ‘Code Grey’ rates 3 months period post opening of PAPU 2007 compared with 3 months period at the same time of year in 2006	Total number of patients restrained decreased from 38 to 17; overall hours in restraint decreased from 197 to 35 h Average hours of mechanical restraint for individual patients dropped from 6.8 to 2.5 h
Cailhol *et al*.^30^	Pre/post‐intervention study	Psychiatric ED Switzerland	Psychiatric emergency arrivals with overdose attempt	254 patients pre‐ education intervention *vs*. 224 patients post‐educational intervention	Assess impact of a staff educational crisis intervention (SECI) educational program: early screening of potential violent behaviour, team procedures, diagnosis and awareness of written guidelines emphasis on regular dialogue between relevant staff members through frequent meetings medical presence during all security interventions debriefing after each intervention	Violent behaviour (VB) rates – VBs defined as any patients requiring intervention of security or restraints 5 months before and after the education intervention	Reduction of violent behaviour from 17.32% to 7.14%; greater in populations with specific diagnoses (major depression, life crisis situations and alcohol or drug intoxication) than borderline personality disorder and psychotic disorder
Cole^26^	Pre/post‐intervention study	Large Metropolitan ED Hawaii, USA	Patients with at least one behavioural health issue	Unspecified	Implementing 4 h training course to adopt BETA project recommendations: best practice techniques for evaluation and triage of agitated patients de‐escalation techniques alternatives to seclusion and restraints staff safety and simulation post‐test and evaluation	Restraint or seclusion usage and hours Data displayed in run chart format over 2.5 years period July 2011 to September 2013, project commenced in February 2012; periods were pre‐training, during training, post‐training with study period ending July 2014	Initially 15–20 incidents requiring restraint/seclusion per month to no incidents by September 2013 Total hours in restraint/seclusion reduced from 38.5 h/month to 0 h/month The data from September 2013 to July 2014 was not displayed, and may not have remained at zero.
Geoffrion *et al*.^31^	Pre/post‐intervention study	Psychiatric ED and Intensive Care Unit (ICU) PsychiPsc Canada	All admissions to psych ICU and ED	Seclusion and restraint episodes Psychiatric ICU 6933 ED 880	Efficacy of Omega training program: teaching of fundamental values (respect, professionalism, accountability, security) and principles (protect oneself, assess the situation, predict behaviour, take the time needed, focus on the person) seven levels of intervention in an intervention pyramid completion of post incident report	Restraint or seclusion usage and hours 5 years	Within ED, no significant differences between pre‐training and post‐training rate of restraint use, but decreasing trend maintained over time Number of restraints per 1000 patient‐days: 3.7 pre‐training *vs*. 1.6 post‐training
Hoffman *et al*.^28^	Retrospective cohort study	Large Paediatric Hospital ED USA	All presentations to paediatric ED	3924 mental health ED visits over 3 years pre‐intervention, 1524 over 1 year during intervention period, 948 during 6 months sustainment period	Effects of an ED agitation care pathway: establish role assignments implement environmental safety measures determine the aetiology and severity of agitation employ verbal de‐escalation techniques	Mean time in physical restraints One year intervention, 6 months maintenance	Mean time in physical restraints reduced from 173 min per episode to 71 min per episode − 59% reduction from baseline. Including 2 outlier cases in June–July 2021 with severe agitation unresponsive to initial medications and requiring multiple medications over several hours. No further cases of prolonged restraint after droperidol became available.
Kelley^37^	Retrospective cohort study 2009–2011	Community Hospital ED USA	All patients in emergency care centre, 90 000 annual visits	650 episodes of activation of team	Effect of introduction of a de‐escalation team which included physicians, nurses, social workers, technicians, security guards, HR personnel, administration personnel, risk management personnel overhead page summons staff at early stages of an escalation physician and nurse assess person security staff in background	Time spent in seclusion or restraint Data from August 2008, June 2009 and August 2009 prior to intervention commencement in December 2009 *vs*. bimonthly data sampling from June	30% of behavioural health hours spent in locked seclusion and some form of restraint required to move the patient to the locked room. Post introduction of procedure restraint and seclusion reduced to less than 1% and 2% of behavioural health hours. Anecdotal reductions in ‘take downs’ from 50% to 80% of the time to six occasions recorded.
Legambi *et al*.^36^	Pre/post‐intervention study	Urban ED USA	All patients in the ED	594 visits pre‐BARS *vs*. 701 visits post‐BARS	Behavioural Activity Rating Scale (BARS)	Restraint use and time 4 months post‐intervention data compared with historic controls from same time of year the previous year	Non‐significant difference in number of restraints used; 20 restraints used pre‐BARS *vs*. 18 restraints used post‐BARS Eight patients kept in restraint >24 h pre‐BARS *vs*. 2 patients kept in restraint post‐BARS
McCurdy *et al*.^32^	Pre/post‐intervention study	Emergency psychiatric area, General Hospital USA	All patients admitted into the emergency psychiatric area	1377 patients at baseline, 1389 post Broset scale, 1513 post installation of the door	Effect of introduction of Broset scale to assess potential violence Effect of a design change that reduced openness (i.e. installing a door to reduce access to different areas of the unit)	Use of seclusion and restraint Baseline November–December 2011, score only January–February 2012, score + door April–May 2012	Reduction of seclusion/restraint use from 44 incidents and no assault pre‐Broset scale (3.2%) Post‐Broset score: 39 incidents of seclusion/restraint and 1 incident of assault (2.8%) Post‐Broset score and door: 27 incidents post‐door (1.8%), no assaults
Pavlov *et al*.^29^	Pre/post‐intervention study	Urban tertiary care paediatric ED USA	Paediatric patients	1465/month pre‐intervention (mean age 14.3 years) 1545/month post‐intervention (mean age 15.4 years)	Evaluation of an agitation protocol in ED, developed by two paediatric ED physicians and three child and adolescent psychiatrists, based in part on best practices publication of project BETA agitation protocol presented to doctors and nurses and made available on every computer	Physical restraint rate, verbal de‐escalation rate, voluntary medication rate, behavioural response team activation rate Protocol introduced February 2016, next 16 months measured. Compared with prior 12 months period, May 2014–2015	Pre‐intervention 31 behavioural response team activations over 13 months compared with post‐intervention 21 during 16 months. Verbal de‐escalation documented in 53% of cases pre‐intervention and in 77.3% of cases post‐intervention Voluntary pharmacology 3.2% pre‐intervention and 14.3% post‐intervention Physical restraint rate 87.1% pre‐intervention and 81% post‐intervention
Uspal *et al*.^34^	Pre/post‐intervention study	Tertiary care children's hospital ED USA	All patients with mental health diagnosis coding excluding organic, physical substance and ID diagnoses	738 visits pre‐intervention *vs*. 902 visits post‐intervention	Assess the effects of a dedicated Mental Health team which includes a mental health evaluator and a paediatric mental health specialist performs MH evaluations, coordinates care, helps implement behavioural interventions, provides brief psychoeducation to patients and families. Available 24/7 adjusted physical environment of ED to enhance safety	Primary: ED LOS Secondary: security physical intervention, and restrain use Post‐intervention March 2011 to March 2012 Pre‐intervention period March 2010 to March 2011	Significant decrease of mean ED LOS from 332 to 244 min Decrease in security intervention from 2.0% to 0.4% and physical restraint use from 1.7% to 0.1%
Winokur *et al*.^27^	Pre/post‐intervention study	General Emergency Department USA	All patients admitted to the ED	Unspecified	Effects of a nurse‐driven standardised procedure (STP) allows nurses to perform clearly defined actions in specific circumstances emergency nurses received extensive education with 2 h didactic training session	Average time to first medication and use of restraints Data from 2014 aggregated. Intervention first quarter of 2015, comparison data collected quarterly from second quarter of 2015 to first quarter of 2017	Decrease of average time to first medication from 43 min to less than 5 min Use of restraint decreased from 9% to 4.8%; time in restraint reduced from 219 to 142 min
Wong *et al*.^35^	Interrupted time series analysis	Urban academic Hospital USA	All ED patients >16 years	634 578 ED visits Median age 47 years, 48% male, 46% substance or MH history	Design implement and evaluate a team‐based interprofessional code response team intervention to manage patient agitation in the ED design and administrative support phase included team‐based training in agitation management, engagement of stakeholders in design of code response team structured team protocol for ED agitation management team comprised of Inside room: team lead emergency physician, lead security officer, lead nurse, patient care technician, backup officer, second nurse as needed Outside room: outside officers, charge nurse, outside nurses	Rates of physical restraint 5 years study: Standard care pre‐intervention data collection January 2015 to June 2016 Design and admin support phase July 2016 to March 2019 March 2019 Implementation of code response team Post‐implementation data collection April 2019 to August 2021	Restraint rate declined from 1.1% to 0.8% over intervention period – corresponding to a 27.3% proportionate decrease.

Three of the papers did not specify the population size of their study.^25^, ^26^, ^27^ Of the remaining 10 studies, the sample size ranged from 478 to 634 578 ED visits. Two studies involved paediatric patients.^28^, ^29^ The studies were conducted in four countries; the majority were conducted in the USA.

Risk of bias of included studies is shown in Table 4. Overall risk of bias was low for one study (8%) and high for 12 studies (92%). All studies had two groups similar and recruited from the same population (criteria #1), exposure measured similarly to assign people to both exposed and unexposed groups (criteria #2), exposure measured in a reliable way (criteria #3), and complete follow‐up (criteria #9). Collectively, 38% (*n* = 5) identified confounding factors (criteria #4), 15% (*n* = 2) employed strategies to deal with confounding factors (criteria #5), 69% (*n* = 9) measured outcomes in a valid or reliable way (criteria #7), 77% (*n* = 10) reported a sufficient follow‐up time (criteria #8) and 15% (*n* = 2) employed appropriate statistical analyses (criteria #11). Notably, criteria #6 (groups free of outcome at start of study) and criteria #10 (strategies to address incomplete follow up) were not applicable to all studies.

**TABLE 4:** Risk of bias of included studies using JBI Critical Appraisal Checklist for Cohort Studies

	Criteria											
Study	#1	#2	#3	#4	#5	#6	#7	#8	#9	#10	#11	Overall
Braitberg *et al*.^33^	Yes	Yes	Yes	Yes	Yes	N/A	Yes	Yes	Yes	N/A	No	High
Browne *et al*.^25^	Yes	Yes	Yes	No	No	N/A	Yes	Yes	Yes	N/A	No	High
Cailhol *et al*.^30^	Yes	Yes	Yes	No	No	N/A	Unclear	No	Yes	N/A	No	High
Cole^26^	Yes	Yes	Yes	No	No	N/A	Unclear	Yes	Yes	N/A	No	High
Geoffrion *et al*.^31^	Yes	Yes	Yes	No	No	N/A	Yes	Yes	Yes	N/A	Yes	High
Hoffman *et al*.^28^	Yes	Yes	Yes	No	No	N/A	Yes	Yes	Yes	N/A	No	High
Kelley^37^	Yes	Yes	Yes	No	No	N/A	Unclear	Yes	Yes	N/A	No	High
Legambi *et al*.^36^	Yes	Yes	Yes	Yes	No	N/A	Yes	No	Yes	N/A	No	High
McCurdy *et al*.^32^	Yes	Yes	Yes	No	No	N/A	Yes	No	Yes	N/A	No	High
Pavlov *et al*.^29^	Yes	Yes	Yes	Yes	No	N/A	Yes	Yes	Yes	N/A	No	High
Uspal *et al*.^34^	Yes	Yes	Yes	Yes	No	N/A	Yes	Yes	Yes	N/A	No	High
Winokur *et al*.^27^	Yes	Yes	Yes	No	No	N/A	Unclear	Yes	Yes	N/A	No	High
Wong *et al*.^35^	Yes	Yes	Yes	Yes	Yes	N/A	Yes	Yes	Yes	N/A	Yes	Low

#1: Were the two groups similar and recruited from the same population? #2: Were the exposures measured similarly to assign people to both exposed and unexposed groups? #3: Was the exposure measured in a valid and reliable way? #4: Were confounding factors identified? #5: Were strategies to deal with confounding factors stated? #6: Were the groups/participants free of the outcome at the start of the study (or at the moment of exposure)? #7: Were the outcomes measured in a valid and reliable way? #8: Was the follow up time reported and sufficient to be long enough for outcomes to occur? #9: Was follow up complete, and if not, were the reasons to loss to follow up described and explored? #10: Were strategies to address incomplete follow‐up utilised? #11: Was appropriate statistical analysis used?

With regard to the measurement of outcomes, four studies used combined seclusion and restraint rates as a single measure,^26^, ^30^, ^31^, ^32^ whereas seven studies reported restraint rates separately.^25^, ^27^, ^29^, ^33^, ^34^, ^35^, ^36^ Six studies reported time in restraint as an outcome,^25^, ^26^, ^28^, ^31^, ^32^, ^36^ although two of these studies did not report restraint rate.^28^, ^37^

Thirteen studies explored various interventions, which fell into four main categories: an agitation protocol and associated training workshops; introduction of a crisis team; geographically separate assessment units; and an agitation scale alone.

Six studies had interventions based on the implementation of an agitation protocol which included training and workshops for staff to develop skills and change overall attitudes towards patients with behaviours of concern.^26^, ^27^, ^28^, ^29^, ^30^, ^31^ These training programmes followed similar principles emphasising the use of de‐escalation techniques as a first‐line option over coercive measures, treating based on aetiology and holding debriefings for patients and their caregivers.

Four of the six studies demonstrated a significant reduction in restraint. Notable findings included: (i) a reduction in restraint time from 173 to 71 min^28^; (ii) a reduction from 15 to 20 episodes of restraint/seclusion per month to negligible levels over 2.5 years^26^; (iii) a reduction in violent behaviour defined as needing security involvement or physical restraint from 17.32% to 7.14%^30^; (iv) an improvement in documentation of verbal de‐escalation from 53% to 77% (paediatric ED setting)^29^; (v) an increase in voluntary pharmacology from 3.2% to 14.3% (paediatric ED setting)^29^; and (vi) a reduced physical restraint rate from 87% to 81% (paediatric ED setting).^29^

Two studies did not show statistically significant findings. The study by Geoffrion *et al*.,^31^ which introduced the Omega training programme, showed decreased restraint and seclusion usage (3.7 restraints per 1000 patient‐days *vs*. 1.6), but did not meet statistical significance. The last study focused on establishing a nurse‐driven standardised procedure which enabled nurses to initiate medication early for behaviours of concern to assist de‐escalation.^27^ The present study saw a reduction of restraint use from 9% to 4.8%, but did not specify whether this reduction was statistically significant.

Three studies investigated the introduction of a crisis team.^34^, ^35^, ^37^ The teams were designed specifically to respond to and assess patients with behaviours of concern in the ED. The components of the teams varied across the different studies, including physicians, nurses, patient care technicians, social workers and security personnel; one study also brought in the use of a mental health evaluator and a paediatric mental health specialist.^34^ Of these studies, two saw a significant reduction in mechanical restraint rate.^34^, ^35^ The study by Kelley *et al*.^37^ found that the introduction of a crisis team was able to achieve a reduction of seclusion from 37% to less than 2% and restraint to less than 1% of total time spent in ED by mental health‐related patients, but did not clarify if the difference was significant.

Two studies from the same hospital in Australia investigated the use of an assessment unit. Both were geographically separated from the rest of the ED, but functioned quite differently.^25^, ^33^ In 2006, a psychiatric assessment and planning unit^25^ was introduced with a separate governance model, resulting in decreased numbers of ED patients needing intervention. The introduction of a behavioural assessment unit under the governance of ED in 2012,^33^ resulted in a significant reduction in rates of mechanical restraint within the ED. Both studies saw a significant reduction in mechanical restraint rates.^25^, ^33^ Although not addressed overtly by the authors, we speculate that the initial strategy^25^ may have informed and/or impacted the effectiveness of the latter^33^; therefore, possible legacy effects of prior interventions warrant consideration when interpreting these findings.

The remaining two studies explored the introduction of a stand‐alone agitation scale.^32^, ^36^ One of these studies also examined the introduction of a closed door between the waiting room and the rest of the unit as an additional intervention.^32^ Both studies that used an agitation scale saw modest and non‐significant reduction in restraint rates.^32^, ^36^ In contrast, McCurdy *et al*.^32^ noted that the addition of a closed door to reduce free movement in the ward, led to a significant reduction of restraint and seclusion events from 3.0% to 1.8%.

## Discussion

This scoping review collated 13 studies, which have been grouped into four interventions: implementation and training in an agitation protocol, introduction of a crisis team, creation of an assessment unit, and incorporation of an agitation scale alone. Among these categories, training in a de‐escalation protocol, the introduction of a crisis team and an assessment unit all showed good levels of evidence in reducing mechanical restraint rate. All of the studies using these interventions demonstrated reduction of time in restraints or restraint rate, and the majority showed a statistically significant reduction. In contrast, the two studies that utilised an agitation scale alone saw non‐significant reductions in restraint. Although the number of studies was low, the result suggests that solely adding an assessment tool may not adequately prepare staff to manage aggression and escalation differently. Notably, only one study was considered low risk of bias.

The evidence base for interventions to reduce mechanical restraint rate has been explored more thoroughly in psychiatric inpatient settings than it has in the ED. Although actual evidence base for interventions remains weak,^18^ there are two clearly structured and well‐utilised conceptual frameworks which have been utilised to reduce the rate of use of restrictive practices, including mechanical restraint and seclusion, primarily in psychiatry inpatient settings. These are the *Six Core Strategies to Reduce the Use of Seclusion and Restraint* ^17^ and the *Safewards* approaches.^19^ The *Six Core Strategies to Reduce the Use of Seclusion and Restraint* ^17^ are: (i) leadership towards organisational change; (ii) use of data to inform practice; (iii) workforce development; (iv) use of seclusion and restraint prevention tools; (v) consumer roles in inpatient settings; and (vi) debriefing techniques. The *Safewards* approach^19^ includes six domains of originating factors: (i) staff team; (ii) physical environment; (iii) outside hospital; (iv) patient community; (v) patient characteristics; and (vi) regulatory framework. Of note, the Melbourne Social Equity Institute Seclusion and Restraint Project report from 2014^38^ directly adopted the *Six Core Strategies to Reduce the Use of Seclusion and Restraint*.^17^

A review by Scanlan *et al*.^16^ in 2010, again in the psychiatry inpatient setting, identified seven key areas of intervention. Some of these can be implemented in ED with greater ease, such as external review/debriefing, data use, training, consumer and carer involvement, staff ratio/crisis responses and program elements such as staff language. The remaining two interventions of policy and environmental changes are reported to be very effective contributors to the reduction in agitation and subsequent restrictive interventions, but are less easily addressed in the ED setting.

Several key effective interventions identified in this review fit within the broad category of workforce development, such as staff education in de‐escalation and introduction of a crisis team. In terms of inpatient settings, the review by Scanlan *et al*.^16^ incised further into workforce development and reported that both the development of de‐escalation skills and tools to develop attitudinal change were necessary to influence change. As per their findings, without these adjustments to staff culture and attitude, reduction of restraint use was unsuccessful.

Furthermore, among the findings of this review, the creation of assessment units can be understood as environmental interventions. In this scoping review, two studies specifically created entirely new geographical spaces,^20^, ^33^ whereas previous psychiatry inpatient studies modified existing ward environments.^16^, ^39^ Both led to reductions in restrictive interventions. These changes include lowering background noise levels (i.e. number of alarms),^16^ providing sensory modulation supplies to patients,^16^, ^39^ and adjustments to ward routines.^16^ The literature review by Bak *et al*.^1^ found that the most effective intervention was therapy focused on using the patient's surroundings to positively affect their mental health (cognitive milieu therapy). This further supports the premise that environment can impact a person's level of agitation. Although these options may not be feasible within the ED setting, it remains important to explore any opportunities to change the environment in order to decrease the rates of restraint use. Although an agitation scale alone is useful in defining behaviour, it does not empower staff to interact more positively with consumers. This may explain in part why the two studies which introduced a scoring system alone did not find any reduction in restrictive outcomes.

### Implications for clinical practice

The implications for clinical practice to be derived from these collective findings include: (i) consideration of staff development initiatives, with focus on staff attitudinal change as well as de‐escalation skills (the *Safewards* initiative has approached this by developing 10 clear objectives in staff‐patient interaction); (ii) if no specifically trained crisis team exists, then this could be developed; and (iii) where possible the reduction of environmental stimulus, ideally via introduction of a purpose‐specific, quiet and separate space. In conjunction with our local knowledge of EDs and clinical systems, these findings suggest that a top‐down, policy and practice‐driven focus on attitudinal change may be useful within the rapidly moving and demanding ED environment.

### Strengths and limitations

The strengths of this review include the breadth of the search strategy, the use of at least two reviewers and the evaluation of risk of bias for each study. Despite this, the scoping literature review has several limitations. First, although the interventions for each of the studies were categorised into four types, in reality, each of these interventions was not uniform. For example, whereas both the BETA recommendations^26^, ^29^ and OMEGA training program^31^ were structured based on the *Six Core Strategies to Reduce the Use of Seclusion and Restraint*,^17^ there were still some differences in the standardised procedure (Table 3). Second, the outcomes reported by each study differed greatly with some reporting seclusion and restraint rates as a single outcome. This is particularly important in Australia, as seclusion rates halved between 2009 and 2022, whereas rates of physical and mechanical restraint have remained largely unchanged in the same time period.^40^ This suggests that across Australia, hospitals have been successful in decreasing seclusion rates, but have had difficulty doing the same for restraint rates. This highlights the need to differentiate between the two outcomes so that interventions that successfully reduce mechanical restraint use are found. Third, the overall risk of bias in included studies tended to be high. Specifically, few studies identified and addressed confounding factors, and few employed appropriate statistical analyses such as interrupted time‐series analysis. Nonetheless, this review adds to an understanding of effective methods for reducing restrictive interventions in the ED setting.

## Conclusion

This review identified a number of interventions with differing degrees of effectiveness in reducing outcomes associated with mechanical restraint in the ED setting. Evidence supports further exploration of interventions that include: designing an agitation guideline; training staff in assessment, attitudinal and de‐escalation skills; addition of a crisis team; and environmental changes in the form of adding a dedicated clinical space. Notably, introducing an agitation assessment tool alone appeared limited in effectiveness. Although some strategies identified in this review may reduce mechanical restraint in the ED setting, further high‐quality studies are needed before definitive conclusions may be drawn.

### Author contributions

DJL, JH and JL designed the study. JL created the search strategy with advice from both DJL and JH. DJL and JL screened for appropriate studies for the literature review with JH monitoring the progress. PJO assessed the study risk of bias. JL, DJL, PJO, and JH reviewed, revised and approved the final manuscript.

### Competing interests

None declared.

## Data Availability

The data that support the findings of the present study are available from the corresponding author upon reasonable request.

## References

[1] Bak J , Brandt‐Christensen M , Sestoft DM , Zoffmann V . Mechanical restraint – which interventions prevent episodes of mechanical restraint? – a systematic review. Perspect. Psychiatr. Care 2012; 48: 83–94. 21967236 10.1111/j.1744-6163.2011.00307.x

[2] Mohr WK , Petti TA , Mohr BD . Adverse effects associated with physical restraint. Can. J. Psychiatry 2003; 48: 330–337. 12866339 10.1177/070674370304800509

[3] De Berardis D , Ventriglio A , Fornaro M *et al*. Overcoming the use of mechanical restraints in psychiatry: a new challenge in the everyday clinical practice at the time of COVID‐19. J. Clin. Med. 2020; 9: 3774. 33238428 10.3390/jcm9113774 PMC7700144

[4] Kersting XAK , Hirsch S , Steinert T . Physical harm and death in the context of coercive measures in psychiatric patients: a systematic review. Front. Psych. 2019; 10: 400. 10.3389/fpsyt.2019.00400 PMC6580992 31244695

[5] Scalise C , Cordasco F , Sacco MA , Aquila VR , Ricci P , Aquila I . Hospital restraints: safe or dangerous? A case of hospital death due to asphyxia from the use of mechanical restraints. Int. J. Environ. Res. Public Health 2022; 19: 8432. 35886284 10.3390/ijerph19148432 PMC9322702

[6] Zun LS , Downey LVA . Level of agitation of psychiatric patients presenting to an emergency department. Prim. Care Companion J. Clin. Psychiatry 2008; 10: 108–113. 18458724 10.4088/pcc.v10n0204 PMC2292436

[7] Mayers P , Keet N , Winkler G , Flisher AJ . Mental health service users' perceptions and experiences of sedation, seclusion and restraint. Int. J. Soc. Psychiatry 2010; 56: 60–73. 20053723 10.1177/0020764008098293

[8] Tingleff EB , Bradley SK , Gildberg FA , Munksgaard G , Hounsgaard L . ‘Treat me with respect’. A systematic review and thematic analysis of psychiatric patients' reported perceptions of the situations associated with the process of coercion. J. Psychiatr. Ment. Health Nurs. 2017; 24: 681–698. 28665512 10.1111/jpm.12410

[9] Anderson E , Mohr DC , Regenbogen I *et al*. Influence of organizational climate and clinician morale on seclusion and physical restraint use in inpatient psychiatric units. J. Patient Saf. 2021; 17: 316–322. 33871417 10.1097/PTS.0000000000000827

[10] Butterworth H , Wood L , Rowe S . Patients' and staff members' experiences of restrictive practices in acute mental health in‐patient settings: systematic review and thematic synthesis. BJPsych Open 2022; 8: e178. 36200350 10.1192/bjo.2022.574 PMC9634587

[11] Brophy LM , Roper CE , Hamilton BE , Tellez JJ , McSherry BM . Consumers and their supporters' perspectives on poor practice and the use of seclusion and restraint in mental health settings: results from Australian focus groups. Int. J. Ment. Health Syst 2016; 10: 6. 26855669 10.1186/s13033-016-0038-x PMC4744440

[12] O'Donovan D , Boland C , Carballedo A . Current trends in restrictive interventions in psychiatry: a European perspective. BJPsych Adv 2023; 29: 274–282.

[13] Social Care, Local Government and Care Partnership Directorate . Positive and Proactive Care: Reducing the Need for Restrictive Interventions. London: Department of Health, 2014.

[14] National Mental Health Commission . Reducing Restrictive Practices. Canberra: Australian Government, 2023.

[15] Engage Victoria . Victoria's Strategy Towards Elimination of Seclusion and Restraint. Melbourne: Victorian State Government, 2023.

[16] Scanlan JN . Interventions to reduce the use of seclusion and restraint in inpatient psychiatric settings: what we know so far a review of the literature. Int. J. Soc. Psychiatry 2010; 56: 412–423. 19617275 10.1177/0020764009106630

[17] National Association of State Mental Health Program Directors . Six Core Strategies to Reduce Seclusion and Restraint Use. Alexandria, VA: National Association of State Mental Health Program Directors, 2008.

[18] Bowers L , Alexander J , Bilgin H *et al*. Safewards: the empirical basis of the model and a critical appraisal. J. Psychiatr. Ment. Health Nurs. 2014; 21: 354–364. 24460906 10.1111/jpm.12085 PMC4237197

[19] Bowers L . Safewards: a new model of conflict and containment on psychiatric wards. J. Psychiatr. Ment. Health Nurs. 2014; 21: 499–508. 24548312 10.1111/jpm.12129 PMC4237187

[20] Health and Human Services . Safewards Handbook: Training and Implementation Resource for Safewards in Victoria [Internet]. Melbourne: Victorian State Government, 2016. [Cited 11 Jun 2024.] Available from URL: https://www.health.vic.gov.au/sites/default/files/migrated/files/collections/policies-and-guidelines/s/safewards-victoria-handbook-2016.pdf

[21] Tricco AC , Lillie E , Zarin W *et al*. PRISMA extension for scoping reviews (PRISMA‐ScR): checklist and explanation. Ann. Intern. Med. 2018; 169: 467–473. 30178033 10.7326/M18-0850

[22] Munn Z , Peters MDJ , Stern C , Tufanaru C , McArthur A , Aromataris E . Systematic review or scoping review? Guidance for authors when choosing between a systematic or scoping review approach. BMC Med. Res. Methodol. 2018; 18: 143. 30453902 10.1186/s12874-018-0611-x PMC6245623

[23] Page MJ , McKenzie JE , Bossuyt PM *et al*. The PRISMA 2020 statement: an updated guideline for reporting systematic reviews. BMJ 2021; 372: n71. 33782057 10.1136/bmj.n71 PMC8005924

[24] Aromataris E , Munn Z , eds. JBI Manual for Evidence Synthesis. JBI, 2020.

[25] Browne V , Knott J , Dakis J *et al*. Improving the care of mentally ill patients in a tertiary emergency department: development of a psychiatric assessment and planning unit. Australas. Psychiatry 2011; 19: 350–353. 21879869 10.3109/10398562.2011.579612

[26] Cole R . Reducing restraint use in a trauma center emergency room. Nurs. Clin. North Am. 2014; 49: 371–381. 25155536 10.1016/j.cnur.2014.05.010

[27] Winokur EJ , Loucks J , Raup GH . Use of a standardized procedure to improve behavioral health patients' care: a quality improvement initiative. J. Emerg. Nurs. 2018; 44: 26–32. 28802869 10.1016/j.jen.2017.07.008

[28] Hoffmann JA , Pergjika A , Liu L *et al*. Standardizing and improving care for pediatric agitation management in the emergency department. Pediatrics 2023; 152: e2022059586. 37317809 10.1542/peds.2022-059586 PMC10312235

[29] Pavlov H , Santillanes G , Claudius I . Decline in pediatric emergency department behavioral team activations after institution of an agitation protocol. Pediatr. Emerg. Care 2021; 37: e170–e173. 33780411 10.1097/PEC.0000000000002403

[30] Cailhol L , Allen M , Moncany AH *et al*. Violent behavior of patients admitted in emergency following drug suicidal attempt: a specific staff educational crisis intervention. Gen. Hosp. Psychiatry 2007; 29: 42–44. 17189744 10.1016/j.genhosppsych.2006.10.007

[31] Geoffrion S , Goncalves J , Giguère CÉ , Guay S . Impact of a program for the management of aggressive behaviors on seclusion and restraint use in two high‐risk units of a mental health institute. Psychiatry Q. 2018; 89: 95–102. 10.1007/s11126-017-9519-6 28500477

[32] McCurdy JM , Haliburton JR , Yadav HC *et al*. Case study: design may influence use of seclusion and restraint. HERD 2015; 8: 116–121. 25929476 10.1177/1937586715575905

[33] Braitberg G , Gerdtz M , Harding S , Pincus S , Thompson M , Knott J . Behavioural assessment unit improves outcomes for patients with complex psychosocial needs. Emerg. Med. Australas. 2018; 30: 353–358. 29219242 10.1111/1742-6723.12905

[34] Uspal NG , Rutman LE , Kodish I , Moore A , Migita RT . Use of a dedicated, non–physician‐led mental health team to reduce pediatric emergency department lengths of stay. Acad. Emerg. Med. 2016; 23: 440–447. 26806468 10.1111/acem.12908

[35] Wong AH , Ray JM , Cramer LD *et al*. Design and implementation of an agitation code response team in the emergency department. Ann. Emerg. Med. 2022; 79: 453–464. 34863528 10.1016/j.annemergmed.2021.10.013 PMC9038629

[36] Legambi TF , Doede M , Michael K , Zaleski M . A quality improvement project on agitation management in the emergency department. J. Emerg. Nurs. 2021; 47: 390–399. 33648736 10.1016/j.jen.2021.01.005

[37] Kelley EC . Reducing violence in the emergency department: a rapid response team approach. J. Emerg. Nurs. 2014; 40: 60–64. 23142097 10.1016/j.jen.2012.08.008

[38] Melbourne Social Equity Institute . Seclusion and Restraint Project: Report [Internet]. Melbourne: University of Melbourne, 2014. [Cited 2024 Jun 11.]. Available from URL: https://socialequity.unimelb.edu.au/__data/assets/pdf_file/0017/2004722/Seclusion‐and‐Restraint‐report.PDF

[39] Väkiparta L , Suominen T , Paavilainen E , Kylmä J . Using interventions to reduce seclusion and mechanical restraint use in adult psychiatric units: an integrative review. Scand. J. Caring Sci. 2019; 33: 765–778. 31058332 10.1111/scs.12701

[40] Australian Institute of Health and Welfare . Seclusion and Restraint in Mental Health Care [Internet]. Canberra: Australian Government, 2024. Available from URL: https://www.aihw.gov.au/mental‐health/topic‐areas/seclusion‐and‐restraint

